# Human papillomavirus E2 proteins suppress innate antiviral signaling pathways

**DOI:** 10.3389/fimmu.2025.1555629

**Published:** 2025-04-08

**Authors:** Jin-Xin Li, Jing Zhang, Cheng-Hao Li, Qing Zhang, Beihua Kong, Pei-Hui Wang

**Affiliations:** ^1^ Department of Infectious Disease and Hepatology, The Second Hospital of Shandong University, Cheeloo College of Medicine, Shandong University, Jinan, Shandong, China; ^2^ Key Laboratory for Experimental Teratology of Ministry of Education and Advanced Medical Research Institute, Cheeloo College of Medicine, Shandong University, Jinan, Shandong, China; ^3^ Department of Obstetrics and Gynecology, Qilu Hospital, Shandong University, Jinan, China; ^4^ Gynecologic Oncology Key Laboratory of Shandong Province, Qilu Hospital, Shandong University, Jinan, China

**Keywords:** HPV E2, persistent infection, interferons, immune evasion, IRF3

## Abstract

Human papillomavirus (HPV) is a major cause of cancers and benign lesions. High-risk (HR) types, including HPV16 and HPV18, are strongly implicated in cervical and other malignancies, while low-risk (LR) types, such as HPV11, are predominantly associated with benign conditions. Although the immune evasion of HPV oncoproteins E6 and E7 are extensively studied, the immunomodulatory functions of the E2 protein remain poorly underexplored. This study elucidates the role of HPV11 and HPV16 E2 proteins in modulating innate immune responses, focusing on their interaction with key innate antiviral signaling pathways. We demonstrate that HPV11 and HPV16 E2 proteins effectively suppress the activation of pivotal antiviral signaling pathways, including RIG-I/MDA5-MAVS, TLR3-TRIF, cGAS-STING, and JAK-STAT. Mechanistic analyses reveal that E2 proteins interact with the core components of type I interferon (IFN)-inducing pathways, inhibiting IRF3 phosphorylation and nuclear translocation, thereby attenuating IFN expression. Additionally, E2 disrupts the JAK-STAT signaling cascade by preventing the assembly of the ISGF3 complex, comprising STAT1, STAT2, and IRF9, ultimately inhibiting the transcription of interferon-stimulated genes (ISGs). These findings underscore the broader immunosuppressive role of HPV E2 proteins, complementing the well-established immune evasion mechanisms mediated by E6 and E7. This work advances our understanding of HPV-mediated immune evasion and positions the E2 protein as a promising target for therapeutic strategies aimed at augmenting antiviral immunity in HPV-associated diseases.

## 1 Introduction

Human papillomavirus (HPV) represent a family of double-stranded DNA viruses that predominantly infect epithelial cells (1), classified into high-risk (HR) and low-risk (LR) types according to their oncogenic potential (2). HR HPV types, including HPV16 and HPV18, are causally linked to cervical, anogenital, and oropharyngeal cancers, with HPV16 alone accounting for nearly 50% of cervical cancer cases worldwide (3, 4). Conversely, LR types, such as HPV11, are primarily associated with benign proliferative lesions, including genital warts (5, 6). Despite their differing oncogenic potential, both HR and LR HPV types employ convergent strategies to evade host immune defenses, enabling persistent infection—a critical determinant of HPV-associated disease progression (7). Persistent HPV infection is pivotal in cancer development, driven by viral oncoproteins E6 and E7, which orchestrate the disruption of host cellular processes, notably immune evasion (8, 9). While the majority of HPV infections are resolved by the host immune system, approximately 10% to 15% persist (10, 11), resulting in chronic infection and elevated oncogenic risk (7). The capacity of HPV to evade innate immune surveillance is fundamental to its persistence and subsequent oncogenic progression (12).

The innate immune system constitutes the primary defense against viral infections, employing pattern recognition receptors (PRRs), including Toll-like receptor 3 (TLR3) (13, 14), RIG-I-like receptors (RLRs) (15, 16), and cyclic GMP-AMP synthase (cGAS) (17–20) to recognize viral elements and activate antiviral responses. Endosomally localized TLR3 detects viral double-stranded RNA, initiating the TRIF-dependent signaling cascade that activates TANK-binding kinase 1 (TBK1), leading to the phosphorylates of interferon regulatory factor 3 (IRF3) (21, 22). Cytoplasmic RLRs, including retinoic acid-inducible gene-I (RIG-I) and melanoma differentiation-associated protein 5 (MDA5), recognize viral RNA and activate TBK1 and IRF3 via mitochondrial antiviral signaling protein (MAVS) (15, 16, 23, 24). Cytosolic viral DNA is sensed by cGAS, which synthesizes the second messenger cyclic GMP-AMP (cGAMP) (17, 19, 20, 25, 26). cGAMP subsequently binds to stimulator of interferon genes (STING), prompting its translocation to the Golgi, where it activates TBK1 to phosphorylate IRF3 (27, 28). These pathways converge on TBK1 and IRF3, critical signaling nodes responsible for induction of interferons (IFNs) (29, 30). IFNs subsequently engage their cognate receptors, activating Janus kinases (JAKs) and driving the phosphorylation of signal transducer and activator of transcription (STAT) proteins. STAT1, STAT2, and IRF9 assemble into the ISGF3 complex, which translocates to the nucleus to activate interferon-stimulated genes (ISGs), thereby fortifying antiviral defense (31, 32).

HPV encodes a limited repertoire of viral proteins, notably E5, E6, and E7, which actively suppress host antiviral responses by interfering with the innate immune signaling pathways. The E5 protein disrupts TLR3-TRIF signaling, thereby attenuating IFN production, whereas E6 associates with TRIM25 and USP15 to destabilize TRIM25, effectively impairing RIG-I signaling (33). HPV16 E7 silences RIG-I expression via SUV39H1-mediated epigenetic modifications and directly inhibits STING in the cGAS-STING pathway, hereby obstructing cellular responses to viral DNA (9, 34). HPV16 E6 and E7 further inhibit the JAK-STAT pathway by downregulating STAT1 expression and preventing its phosphorylation and nuclear translocation, thereby disrupting the ISGF3 complex formation and suppressing ISG transcription (35, 36). Additionally, E6 inhibits STAT2 activation through interactions with Tyk2 (37), whereas E7 disrupts the interaction between IRF9 and STAT1/STAT2, further impeding ISGF3 assembly (38, 39). These inhibitory effects on the JAK-STAT pathway enable HPV to evade host antiviral defenses, facilitating persistent infection. Collectively, these strategies underpin HPV’s ability to evade host immune surveillance and establish long-term persistence.

In addition to E6 and E7, other HPV proteins, including E1 and E2, play essential roles in the viral life cycle. The E2 protein, in particular, governs viral gene expression by binding to the viral upstream regulatory region (URR) and ensures the stable maintenance of the viral genome within host cells (40, 41). While the roles of E5, E6, and E7 in immune evasion are well-defined, the immunomodulatory functions of E2 remain poorly elucidated. Emerging evidence suggests that HPV E2 can downregulate critical immune components, including MDA5 (42), STING and IFN-κ, while concurrently suppress ISG expression (43, 44). Additional studies have shown that HPV16 E2 represses interferon-inducible genes in HPV-positive tumors, further underscoring its role in subverting host immune responses. Despite these insights, the role of E2 in immune regulation remains poorly understood, and the precise mechanisms by which HPV E2 modulates host antiviral pathways have yet to be fully elucidated. Given its critical role in viral gene regulation and genome maintenance, further investigation into its potential immunomodulatory functions is essential for understanding HPV persistence and pathogenesis.

## 2 Materials and methods

### 2.1 Cell lines

HEK293T, HEK293TT, HeLa, Vero, and L929 cell lines were obtained from the American Type Culture Collection (ATCC). The cells were maintained in Dulbecco’s Modified Eagle Medium (DMEM; Gibco, USA) supplemented with 10% fetal bovine serum (FBS; Gibco, USA), 100 U/mL penicillin, and 100 μg/mL streptomycin. All cells were incubated in a humidified atmosphere at 37°C with 5% CO_2_.

### 2.2 Plasmids and transfection

Plasmids encoding RIG-I, RIG-IN, MDA5, MAVS, TRIF, STING, TBK1, IKKϵ, and IRF3-5D were constructed in our previous studies (45, 46). The luciferase reporter plasmids of IFNβ (IFNβ-Luc) and ISGs (ISRE-Luc) were described in earlier studies (45, 46). Plasmids containing the full-length genomes of HPV16 and HPV11 were obtained from ATCC. DNA fragments encoding HPV11 and HPV16 E2 were synthesized (General Biology, China) and subsequently subcloned into pXJ2-Flag and pXJ2-Myc expression vectors. pXJ2 is a eukaryotic expression vector capable of driving high-level expression of target proteins in mammalian cells. Plasmids expressing STAT1, STAT2, and IRF9 were also constructed using pXJ2 vectors. To assess subcellular localization of E2 protein, pDsRed2-Mito, pDsRed2-ER, and pEYFP-Golgi plasmids were purchased from Clontech (USA). Transfections were conducted using Polyethylenimine “MAX” (Polysciences, USA) for HEK293T cells and Lipofectamine 3000 (Invitrogen, USA) for HeLa cells. Poly(I:C) was delivered using Lipofectamine 2000 (Invitrogen). All transfections followed the manufacturer’s protocols.

### 2.3 Antibodies and reagents

Primary antibodies utilized in this study included: mouse anti-Myc (19C2), mouse anti-GAPDH (3B3), mouse anti-IRF3 (CY5779), mouse anti-TBK1 (CY5145), and mouse anti-Lamin B1 (AB0054) (Abways, USA); rabbit anti-Myc (71D10), rabbit anti-pIRF3 (4D46), and rabbit anti-pTBK1 (D52C2) (Cell Signaling Technology, USA); rabbit anti-Flag (Immunoway, USA); and mouse anti-Flag M2 (Sigma-Aldrich, USA). Secondary antibodies included horseradish peroxidase (HRP)-conjugated anti-mouse IgG (AB0102) and anti-rabbit IgG (AB1010) (Abways, USA), along with fluorescence-labeled Alexa Fluor antibodies—Alexa Fluor 488 goat anti-rabbit IgG, Alexa Fluor 594 goat anti-rabbit IgG, Alexa Fluor 488 goat anti-mouse IgG, and Alexa Fluor 594 goat anti-mouse IgG (Beyotime, China). Anti-Flag magnetic beads were purchased from Abmart (China).

### 2.4 Quantitative Real-Time PCR (RT-qPCR) analysis

Total RNA was extracted using the TRIzol reagent (Invitrogen, USA) in accordance with the manufacturer’s protocol. Reverse transcription was conducted using the HiScript III 1st Strand cDNA Synthesis Kit (Vazyme, China). RT-qPCR assays were performed using the UltraSYBR Mixture (CWBIO) on a Roche LightCycler 96 system. Primers sequences utilized in the study are provided in **Supplementary Table 1**. All experiments were conducted in triplicate, and relative gene expression levels were normalized to GAPDH using the 2^-ΔΔCt^ method.

### 2.5 Dual-luciferase reporter assays

Dual-luciferase reporter assays were performed using the Dual-Luciferase Reporter Assay Kit (Vazyme) following the manufacturer’s protocol. HEK293T cells were co-transfected with Firefly luciferase reporter plasmids (IFNβ-Luc and ISRE-Luc), Renilla luciferase control plasmids (pRL-TK), and the respective protein expression plasmids. After 30 hours of transfection, cells lysates were prepared, and luciferase activity was quantified. Firefly luciferase activity was normalized against Renilla luciferase activity to control for transfection efficiency.

### 2.6 Co-immunoprecipitation and immunoblot analysis

HEK293T cells (3 × 10^6^ cells/flask) were seeded into T25 culture flasks and transfected with plasmids for 36 hours. For co-immunoprecipitation, cells were washed with phosphate-buffered saline (PBS) and lysed in ice-cold lysis buffer (150 mM NaCl, 1% NP-40, 50 mM EDTA, and 50 mM Tris-HCl, pH 7.4), supplemented with protease and phosphatase inhibitors (Sigma-Aldrich). Cell lysates were clarified by centrifugation at 13,000 × g for 15 minutes at 4°C, and protein concentrations were quantified using a BCA assay kit (Beyotime). An aliquot (one-tenth) of the lysate was mixed with 5× SDS loading buffer and boiled at 100°C for 15 minutes for input analysis. The remaining lysate was incubated with anti-Flag magnetic beads (Sigma-Aldrich) at 4°C overnight. The magnetic beads were washed four times with lysis buffer, resuspended in 2× SDS loading buffer, and boiled at 100°C for 10 minutes. For immunoblotting, protein samples were separated via SDS-PAGE, and transferred onto PVDF membranes (Millipore). The membranes were blocked in 5% (w/v) nonfat milk and subsequently incubated with appropriate primary and secondary antibodies. Protein bands were visualized using the SuperSignal chemiluminescent ECL reagent kit (Beyotime).

### 2.7 Immunofluorescence

Immunofluorescence assays were performed as described in previous studies (45, 46). HEK293T cells (1 × 10^5^ cells/well) and HeLa cells (3 × 10^4^ cells/well) were seeded onto glass coverslips, transfected with the indicated plasmids for 20 hours, and subsequently stimulated with virus for 6 hours. The cells were washed with PBS, fixed with 4% paraformaldehyde, permeabilized with 0.2% Triton X-100 in PBS with Tween-20 (PBST), and blocked using an immunofluorescence assay kit (Beyotime) according to the manufacturer’s instructions. After blocking and gentle washing, the cells were incubated with the primary antibodies, followed by fluorescence-conjugated secondary antibodies. Coverslips were mounted onto slides using a mounting medium containing DAPI (Beyotime) for nuclear staining. Images were captured using a Zeiss LSM900 confocal microscope and processed using ImageJ software.

### 2.8 Nuclear and cytoplasmic fractionation

Nuclear and cytoplasmic protein fractions were isolated from HEK293T cells using the Nuclear and Cytoplasmic Protein Extraction Kit (P0027; Beyotime) according to the manufacturer’s protocol. Briefly, HEK293T cells (3 × 10^6^ cells per T25 flask) were seeded and cultured overnight before transfection. Following transfecting with plasmids for 36 hours, cells were stimulated with Sendai virus (SeV) for 6 hours. Cells were washed with PBS and centrifuged at 1,000 × g for 5 minutes. For cytoplasmic proteins extraction, the cell pellets were resuspended in Cytoplasmic Extract Reagent A (P0027-1; Beyotime) supplemented with a protease inhibitor mixture and incubated on ice for 15 minutes with periodic vortexing. Cytoplasmic Extract Reagent B (P0027-2; Beyotime) was subsequently added, followed by vortexing and centrifugation at 13,000 × g for 15 minutes at 4°C. The supernatant was retained as the cytoplasmic fraction. To isolate nuclear protein, the remaining pellet was resuspended in Nuclear Extract Reagent (P0027-3; Beyotime) and incubated on ice for 30 minutes with periodic vortexing. The lysates were centrifuged at 13,000 × g for 15 minutes at 4°C, and the supernatant was collected as the nuclear protein fraction.

### 2.9 Viruses and infection

The viral strains utilized in this study were vesicular stomatitis virus (VSV)-enhanced green fluorescent protein (eGFP), Sendai virus (SeV), murine coronavirus mouse hepatitis virus-A59 (MHV-A59), herpes simplex virus type 1 (HSV), and HPV16 virions. Viral infections were conducted according to established protocols (46–48). Cells were washed with prewarmed serum-free DMEM and incubated with virus preparations diluted in DMEM at the desired multiplicity of infection (MOI) for 1–2 hours. Following the infection period, the virus-containing supernatant was discarded, and the cells were maintained in fresh complete DMEM.

### 2.10 Production of HPV16 virions

Infectious HPV16 virions were generated using a transient transfection method based on established protocols (49). Briefly, HEK293TT cells were seeded in 10-cm culture dishes 24 hours before transfection. The cells were co-transfected with plasmids encoding HPV16 L1 and L2 capsid proteins, along with the full-length HPV16 genome using Lipofectamine 2000 (Invitrogen). Following 48 hours of incubation at 37°C, the cells were harvested, lysed in a custom lysis buffer, and virions were concentrated using the polyethylene glycol (PEG8000) method. The purified virions were used for subsequent infection experiments with HeLa cells.

### 2.11 Virus titration

Virus titers were determined using a plaque assay on Vero cells. Vero cells (1.2 × 10^5^ cells per well) were seeded into 24-well plates 24 hours before infection. Virus samples were serially diluted in serum-free DMEM. The Vero cells were washed with PBS and then exposed to the diluted virus for 1 hour. Following infection, the virus-containing medium was replaced with 0.5% agar overlay prepared in DMEM supplemented with 2% FBS. Once the agar overlay solidified, the cells were incubated for 24 hours and subsequently fixed with a methanol-ethanol solution for 30 minutes. The agar overlay was gently removed, and the cells were stained with 0.05% crystal violet for 15 minutes. Plaques were counted, and virus titers were calculated as plaque-forming units per milliliter (PFU/mL) using standard calculation methods.

### 2.12 Statistics

All data analyses were performed using GraphPad Prism 9 software. Statistical significance was assessed using a two-tailed unpaired Student’s t-test, and results are reported as mean ± standard deviation (SD). A p-value of <0.05 was considered indicative of statistically significant. The levels of significance are denoted as follows: *p < 0.05, ** *p* < 0.01, *** *p* < 0.001, ****p < 0.0001.

## 3 Results

### 3.1 HPV E2 suppresses IFN and inflammatory responses triggered by diverse viral stimuli

**Figure 1:**
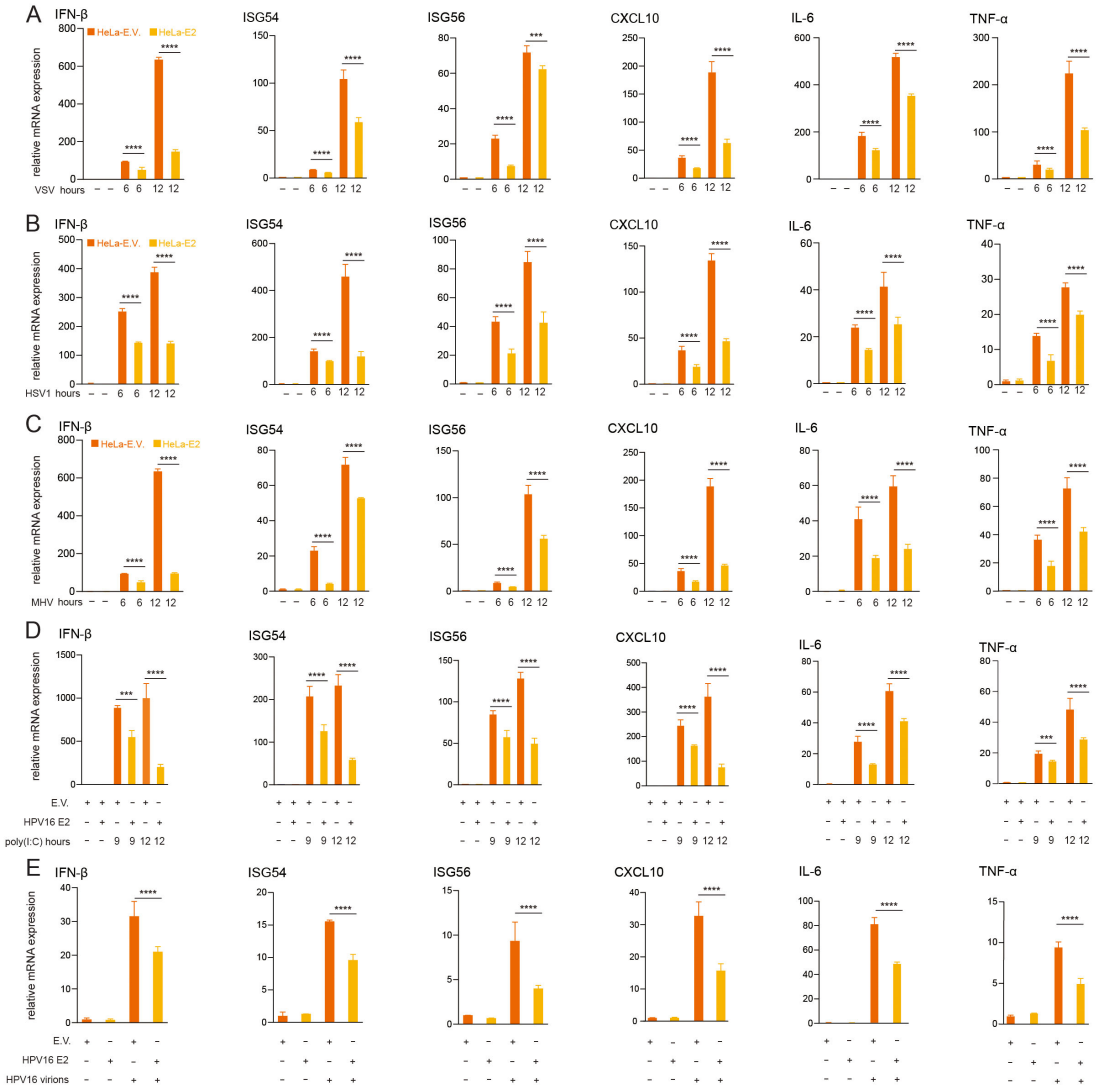
HPV16 E2 suppresses IFN and ISG expression. **(A-C)** HeLa cells stably expressing HPV16 E2 or an empty vector were infected with VSV (MOI=0.1), HSV1 (MOI=1), or MHV (MOI=0.2) for 6 and 12 hours, RNA was extracted and RT-qPCR was performed to quantify mRNA levels of IFN-β, ISG54, ISG56, CXCL10, IL-6 and TNF-α. **(D)** HEK293T cells transfected with plasmid expressing HPV16 E2 or an empty vector were treated with poly(I:C) for 9 and 12 hours, followed by RT-qPCR to measure mRNA levels of IFN-β, ISG54, ISG56, CXCL10, IL-6 and TNF-α. **(E)** HeLa cells stably expressing E2 or an empty vector were infected with HPV16 virions for 24 hours, followed by RT-qPCR to analyze IFN-β, ISG54, ISG56, CXCL10, IL-6 and TNF-α mRNA levels. Data are presented as mean ± SD (n = 3). Statistical significance was analyzed using Student’s t-test (*p < 0.05, **p < 0.01, ***p < 0.001, ****p < 0.0001). E.V., empty vector.

Persistent HR HPV16 infection contributes to cervical cancer and other diseases, with immune evasion playing a pivotal role in sustaining viral persistence. Previous research has established that HPV non-structural proteins, such as E5, E6, and E7, suppress innate antiviral immune responses, facilitating immune evade evasion (9, 43, 50–53). To determine whether HPV16 E2 modulates host innate immunity, we evaluated its impact on virus-induced IFN responses. HeLa cells stably expressing HPV16 E2 or an empty vector were infected with VSV, MHV, and HSV1. RT-qPCR analysis revealed that E2-expressing cells exhibited significantly reduced mRNA levels of IFN-β, ISG54, ISG56, and CXCL10 compared to controls (**Figures 1A–C**). Similarly, HEK293T cells transiently transfected with HPV16 E2 or an empty vector and stimulated with poly(I:C) showed significantly reduced expression of IFN-β and ISGs in E2-expressing cells (**Figure 1D**). Furthermore, infection of HeLa cells stably expressing HPV16 E2 with HPV16 virions, resulted in significantly reduced mRNA levels of IFN-β, ISG54, ISG56, and CXCL10 compared to empty vector controls (**Figure 1E**). To determine whether HPV E2 modulates NF-κB signaling, we examined the expression levels of IL-6 and TNF-α, key target genes of the NF-κB pathway, using RT-qPCR in these samples. Our results showed that HPV E2 significantly reduced IL-6 and TNF-α expression, suggesting that E2 suppresses NF-κB-mediated inflammatory responses (**Figure 1**). These results demonstrate that HPV16 E2 effectively suppresses innate immune responses triggered across various viral stimuli.

### 3.2 HPV E2 facilitates virus replication

**Figure 2:**
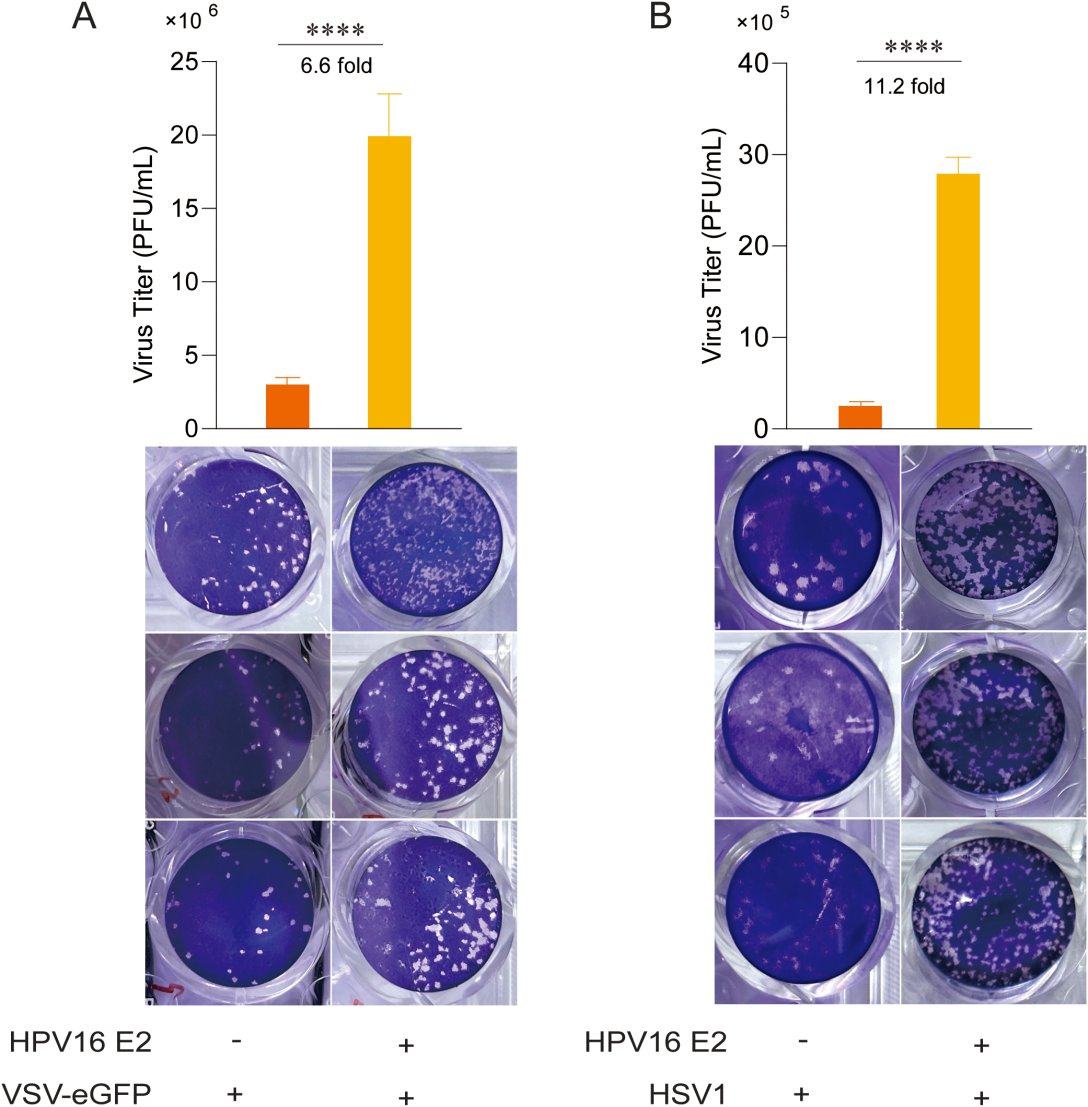
HPV16 E2 facilitates viral replication. **(A, B)** HEK293T cells were co-transfected with plasmids expressing HPV16 E2 or an empty vector. After 24 hours, cells were infected with VSV-eGFP (MOI=0.1) or HSV1 (MOI=1). Culture supernatants were collected 24–36 hours post-infection and analyzed via plaque assays to measure virus titer. Data are shown as mean ± SD from three independent experiments. Statistical significance was analyzed using Student’s t-test (****p < 0.0001).

Following the observation that HPV16 E2 suppresses IFN responses, we investigated its role in viral replication. Due to the host and tissue specificity of HPV replication, surrogate models were employed for these experiments. HEK293T cells overexpressing HPV16 E2 or an empty vector were infected with VSV-eGFP and HSV1 to simulate HPV infection. Plaque assays of culture supernatants demonstrated significantly elevated viral titers in HPV16 E2-expressing cells compared to controls (**Figures 2A, B**). These results suggest that HPV16 E2 promotes viral replication, potentially through the suppression of innate antiviral immune responses.

### 3.3 HPV E2 restrains IFN expression by modulating innate immune signaling pathways

**Figure 3:**
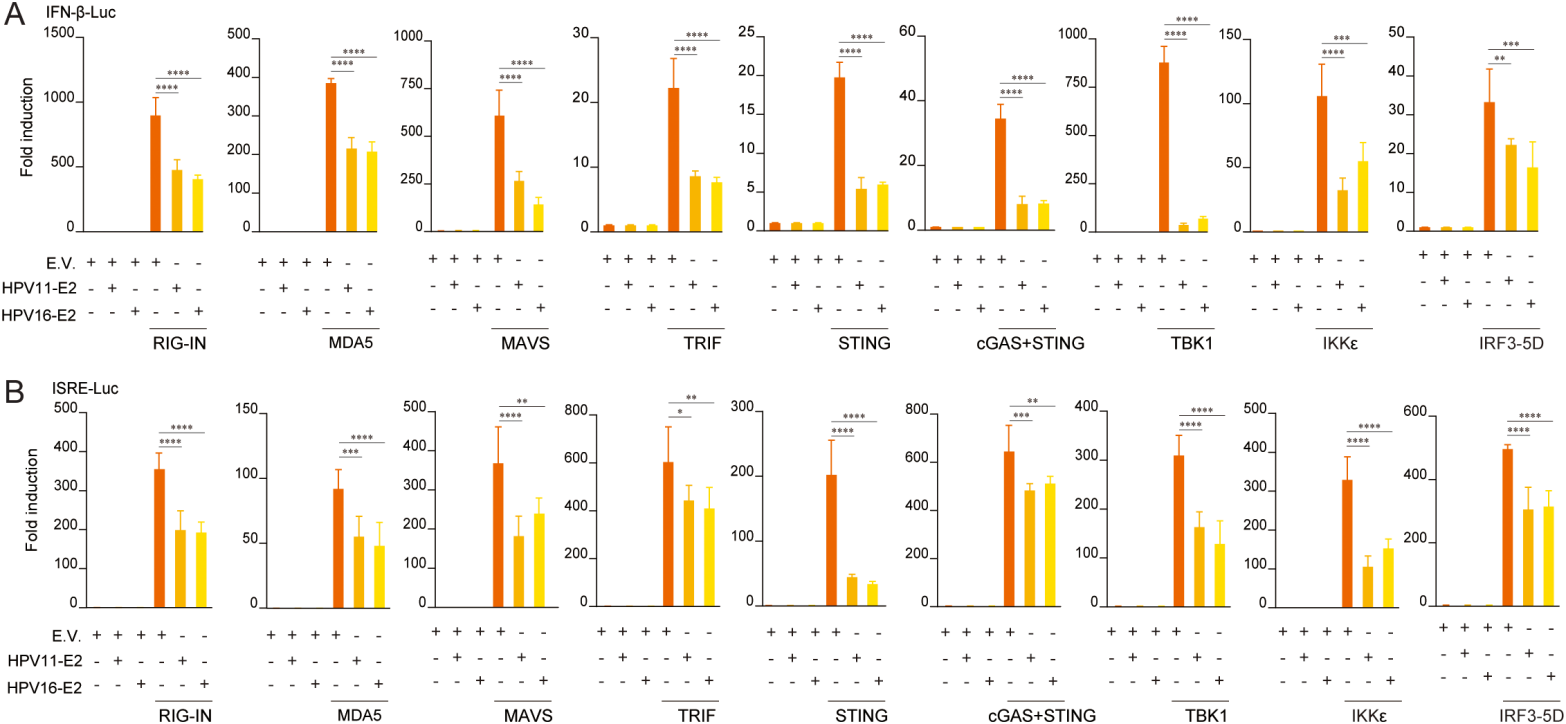
HPV11 and HPV16 E2 inhibit IFN and ISG luciferase reporter activity. **(A, B)** HEK293T cells were co-transfected with plasmids expressing HPV11 E2, HPV16 E2, or an empty vector (E.V.), along with innate immune signaling pathway activators, and IFN-β-Luc **(A)** or ISRE-Luc **(B)** reporters. For all conditions, the empty vector was co-transfected alongside the respective expression plasmids to ensure consistent experimental controls. After 30 hours, luciferase activity was measured 30 hours post-transfection. Experiments were conducted in triplicate. Statistical significance was analyzed using Student’s t-test (*p < 0.05, **p < 0.01, ***p < 0.001, ****p < 0.0001).

**Figure 4:**
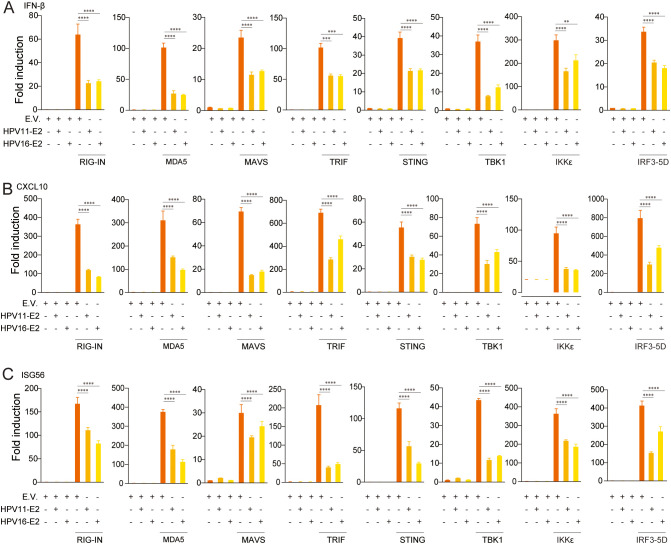
HPV11 and 16 E2 suppress IFN and ISG expression. **(A-C)** HEK293T cells were co-transfected with plasmids expressing HPV11 and HPV16 E2 along with innate immune signaling pathway activators, or an empty vector as control for 24 hours. Control conditions included co-transfection of the empty vector (E.V.) with pathway activators to maintain consistency across experimental groups. Total RNA was extracted and analyzed by RT-qPCR to measure mRNA levels of IFN-β **(A)**, CXCL10 **(B)**, and ISG56 **(C)**. Data represent results from three biological replicates, with SD shown. Statistical significance was analyzed using Student’s t-test (**p < 0.01, ***p < 0.001, ****p < 0.0001).

To elucidate the mechanisms underlying HPV E2-mediated suppression of IFN production, we examined its effects on key antiviral signaling pathways. HEK293T cells were co-transfected with plasmids encoding HPV11 or HPV16 E2 and pathway-specific activators, including RIG-IN, RIG-I, MDA5, MAVS, TBK1, IKKϵ, STING, TRIF, and IRF3-5D, along with luciferase reporters for IFN-β or ISRE activation. Luciferase reporter assays showed that E2 consistently suppressed the activation of both IFN-β and ISRE promoters across all evaluated pathways (**Figures 3A, B**). RT-qPCR analysis further validated that E2 expression significantly decreased mRNA levels of IFN-β, CXCL10, and ISG56 in cells overexpressing these pathway activators (**Figures 4A–C**). These findings indicate that HPV11 and HPV16 E2 broadly suppress innate immune responses by targeting the RIG-I/MDA5, TLR3-TRIF, and cGAS-STING signaling pathways.

### 3.4 HPV E2 interacts with components of innate immune signaling pathways

**Figure 5:**
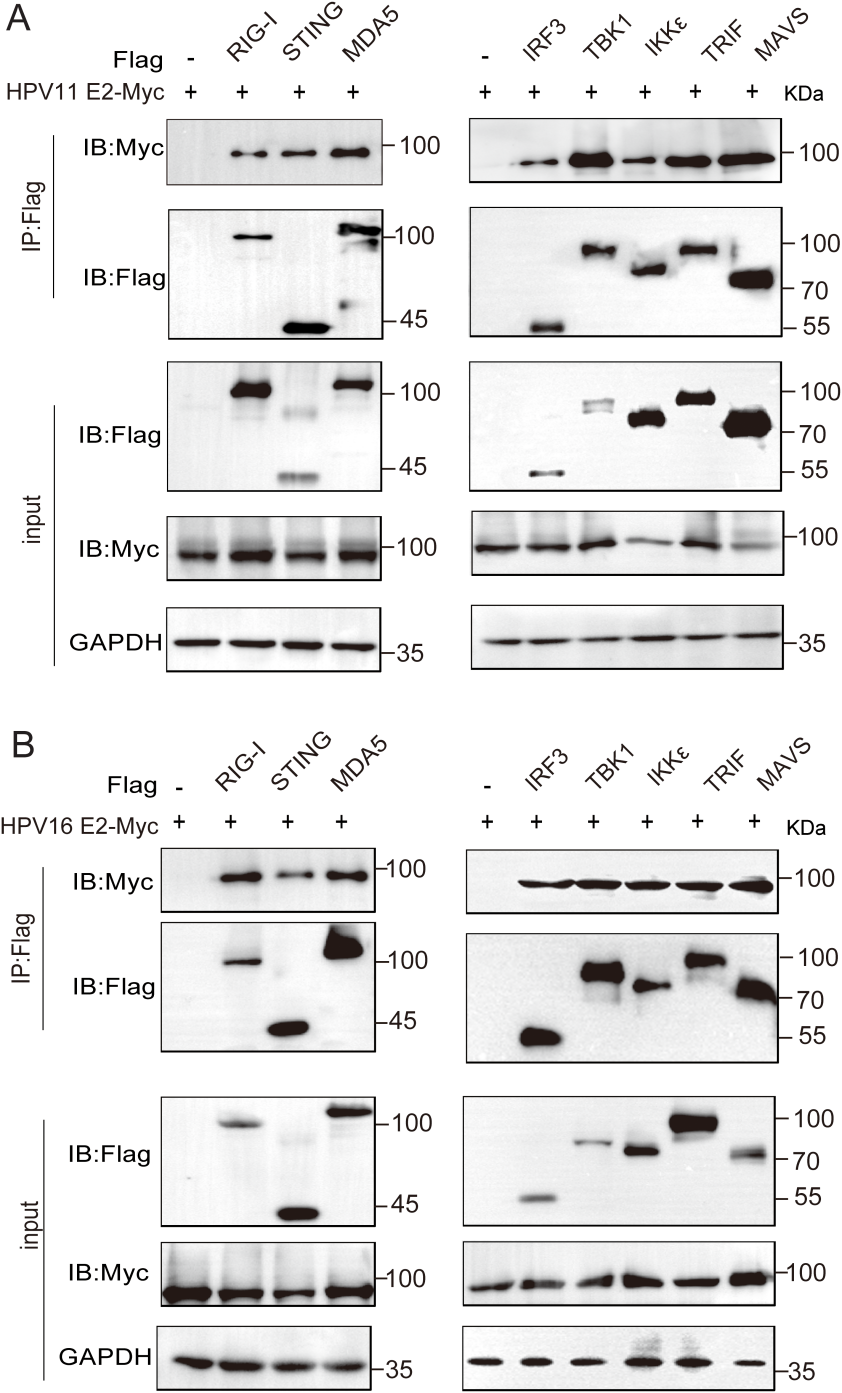
HPV11 and HPV16 E2 interact with RIG-I, MDA5, STING, MAVS, TRIF, TBK1, IKKϵ, and IRF3. **(A, B)** HEK293T cells were co-transfected with plasmids expressing HPV11 E2 **(A)** or HPV16 E2 **(B)**, along with plasmids expressing innate immune signaling proteins. Cells were harvested 36–48 hours post-transfection for co-immunoprecipitation and western blotting were performed.

**Figure 6:**
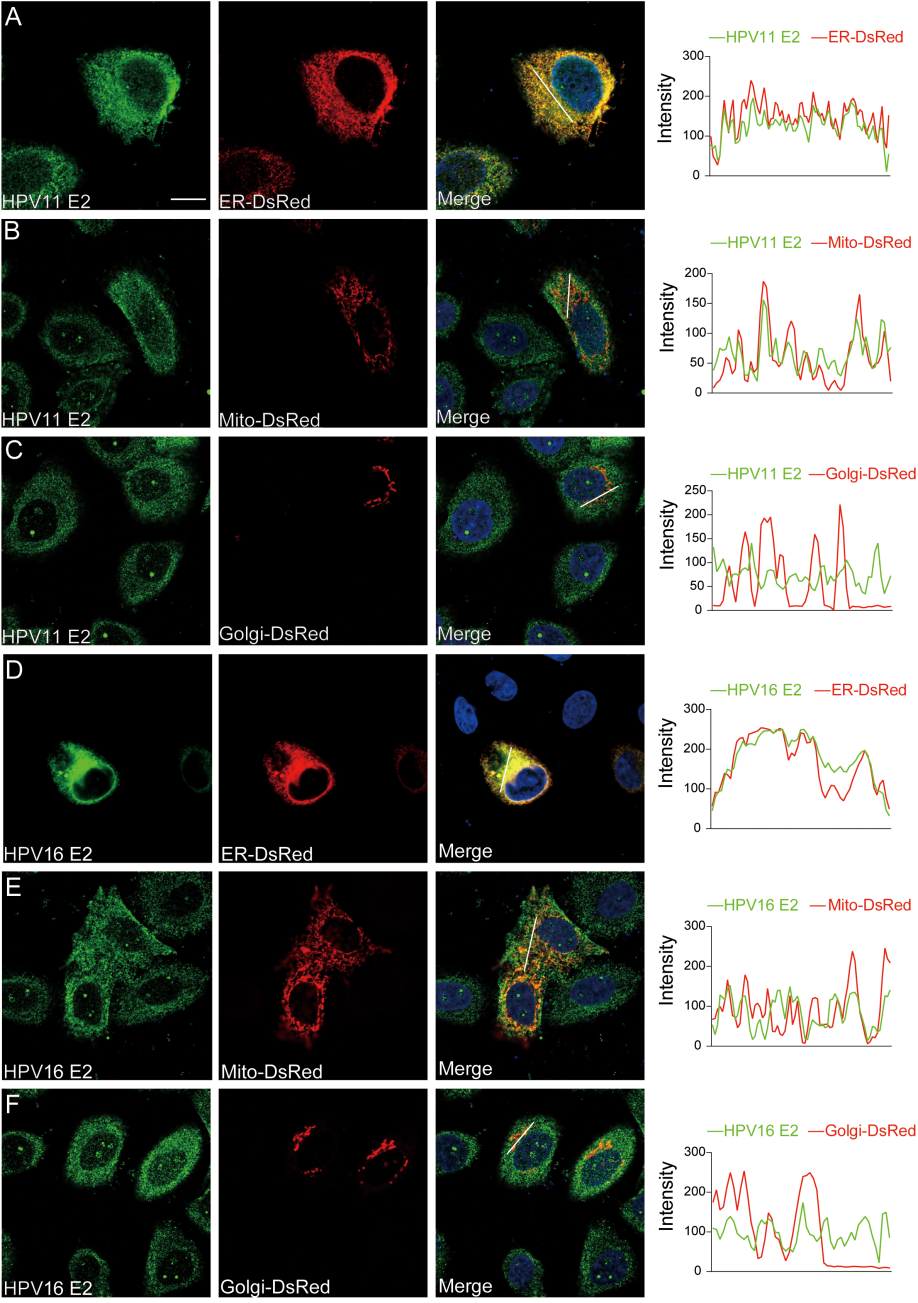
Subcellular localization of HPV11 and HPV16 E2. **(A-F)** HeLa cells were transfected with plasmids expressing HPV11 E2 **(A-C)** or HPV16 E2 **(D-F)** and plasmids encoding organelle marker plasmids for ER, mitochondria, and Golgi. Cells were processed for immunofluorescence using primary and fluorescence-conjugated secondary antibodies. Nuclei was counterstained with DAPI. Scale bar, 10 μm.

**Figure 7:**
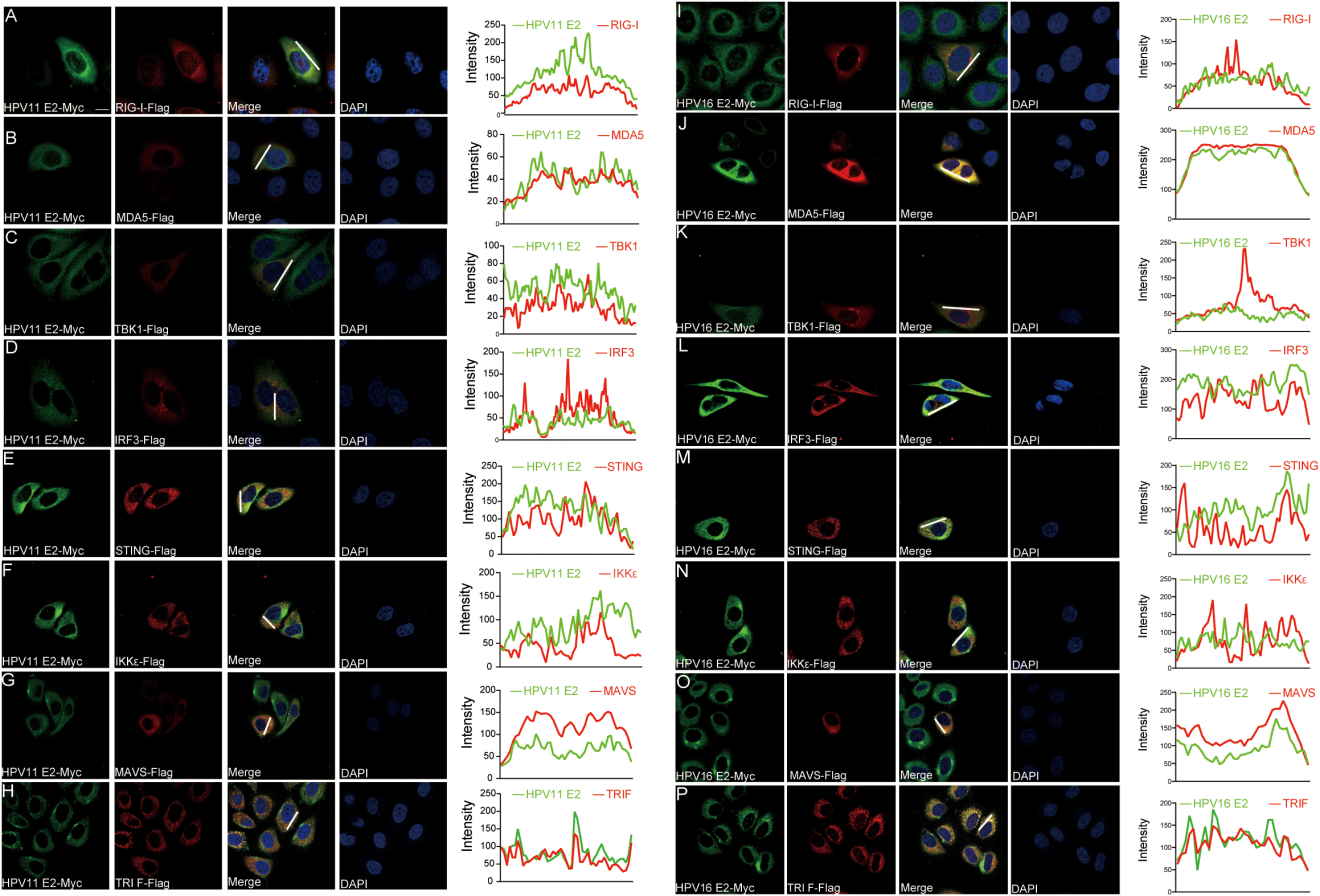
Colocalization of HPV11 and HPV16 E2 with innate immune signaling proteins. **(A-P)** HeLa cells were co-transfected with plasmids expressing HPV11 E2 **(A-H)** or HPV16 E2 **(I-P)**, along with plasmids for innate immune signaling pathway proteins. Cells were fixed 20 hours post-transfection and processed for immunofluorescence. Nuclei was stained with DAPI (blue). Scale bar, 10 μm.

To identify the molecules targets of HPV E2 in IFN suppression, co-immunoprecipitation and confocal microscopy were employed to assess interactions between E2 and key signaling proteins. Co-immunoprecipitation assays show that HPV11 and HPV16 E2 interact with multiple signaling proteins, including RIG-I, MDA5, MAVS, TRIF, STING, IKKϵ, TBK1, and IRF3 (**Figures 5A, B**). Confocal microscopy revealed colocalization of these innate immune signaling proteins and an ER marker with HPV E2 (**Figures 6A–F**, **Figures 7A–P**), suggesting its association with ER-localized signaling complexes. These results indicate that HPV E2 modulates the RLR signaling pathway through interactions with RIG-I, MDA5, and MAVS, and potentially affects TLR3-TRIF signaling via interaction with TRIF. Additionally, HPV11 and HPV16 E2 appear to influence the cGAS-STING pathway by interacting with STING, contributing to the suppression of IFN responses across critical antiviral signaling pathways.

### 3.5 HPV E2 inhibits IRF3 phosphorylation and nuclear translocation

**Figure 8:**
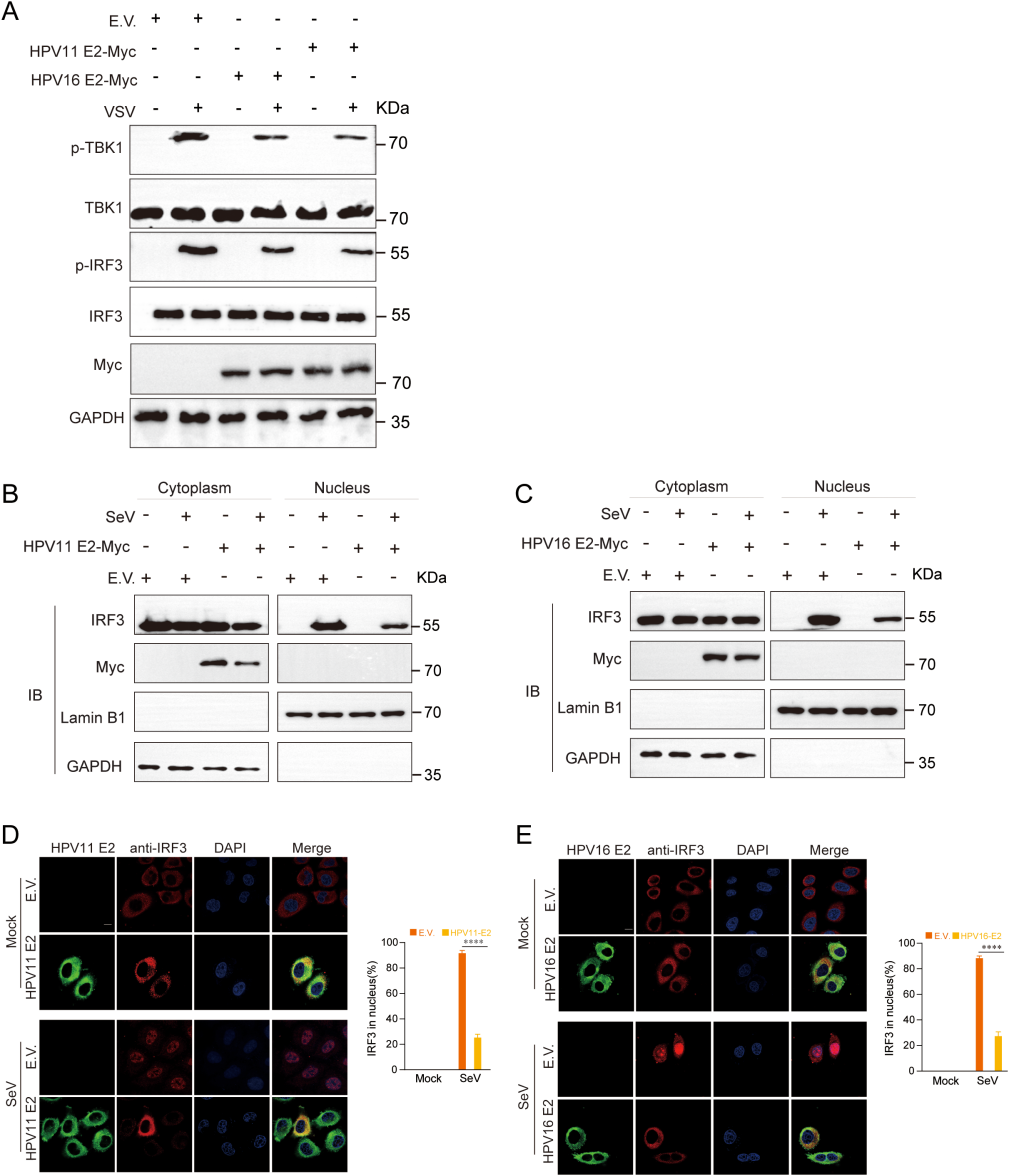
HPV11 and HPV16 E2 inhibit the phosphorylation and nuclear translocation of IRF3. **(A)** HEK293T cells transfected with plasmids expressing HPV11 E2, HPV16 E2, or an empty vector were infected with VSV (MOI=0.1). TBK1 and IRF3 phosphorylation were analyzed by western blotting 24 hours post-transfection. **(B-C)** HEK293T cells overexpressing HPV11 E2 **(B)** or HPV16 E2 **(C)** were infected with SeV (MOI=1). Six hours post-transfection, cells were harvested for nuclear-cytoplasmic fractionation. The nuclear and cytoplasmic fractions were analyzed by western blotting for IRF3, HPV11 E2, HPV16 E2, Lamin B1 (nuclear marker), and GAPDH (cytoplasmic marker). **(D, E)** HeLa cells overexpressing HPV11 E2 **(D)** or HPV16 E2 **(E)** were seeded onto 12 well coverslips overnight and subsequently infected with SeV (MOI=1). Immunofluorescence staining was performed 6 hours post-infection to observe IRF3 localization. Scale bar, 10 μm. The percentage of nuclear-localized IRF3 was quantified based on immunofluorescence results (90-100 cells per group; 3 replicates). Data are shown as mean ± SD (n=3). Statistical significance was analyzed using Student’s t-test (****p < 0.0001). Abbreviations: 11E2, HPV11 E2; 16E2, HPV16 E2.

Phosphorylation and nuclear translocation of IRF3 are pivotal events in the RIG-I/MDA5, TLR3-TRIF, and cGAS-STING pathways that culminate in IFN production (54). Western blot analysis showed that HPV11 and HPV16 E2 proteins markedly suppressed TBK1 and IRF3 phosphorylation in VSV-infected HeLa cells (**Figure 8A**). Nuclear-cytoplasmic fractionation and confocal microscopy further demonstrated that HPV11 and HPV16 E2 proteins inhibited IRF3 nuclear translocation in SeV-infected cells, resulting in its cytoplasmic retention (**Figures 8B–E**). These results indicate that HPV E2 proteins suppress IFN production by blocking IRF3 phosphorylation and nuclear translocation.

### 3.6 HPV E2 disrupts JAK-STAT pathway by inhibiting ISGF3 complex formation

**Figure 9:**
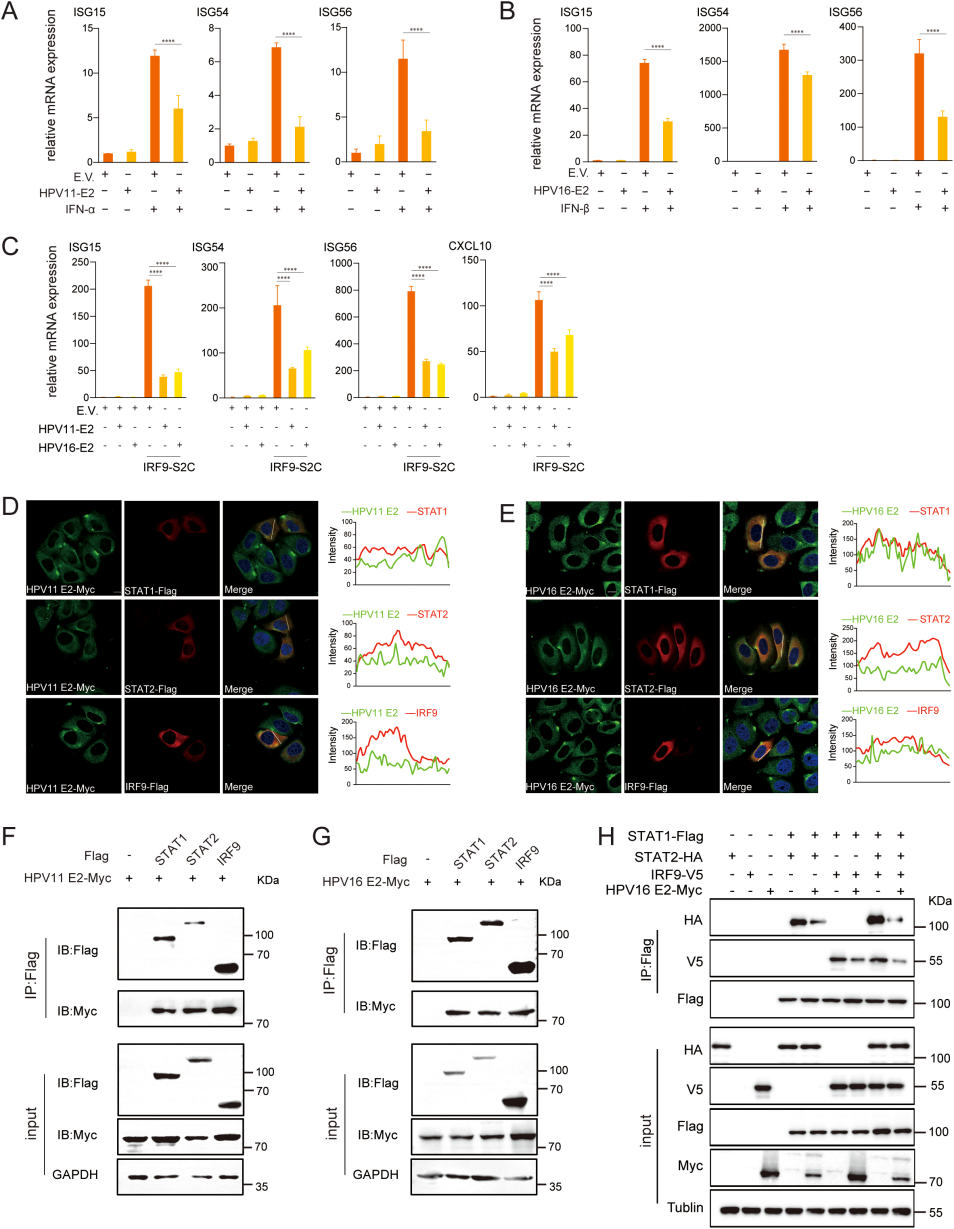
HPV11 and HPV16 E2 inhibit ISGF3 complex formation. **(A, B)** HEK293T cells transfected with plasmids expressing HPV11 E2, HPV16 E2, or an empty vector were stimulated with IFN-α or IFN-β for 6 hours, 24 hours post-transfection. mRNA levels of ISG15, ISG54, and ISG56 were quantified by RT-qPCR. Data are shown as mean ± SD (n=3). Statistical significance was analyzed using Student’s t-test (****p < 0.0001). **(C)** HEK293T cells were transfected with plasmids expressing HPV11 E2, HPV16 E2, along with the IRF9-S2C plasmid, as indicated. Total RNA was extracted 24 hours post-transfection, reverse transcribed, and analyzed for mRNA levels of ISG15, ISG54, ISG56, and CXCL10 by RT-qPCR. Data are shown as mean ± SD (n=3). Statistical significance was analyzed using Student’s t-test (****p < 0.0001). **(D, E)** HeLa cells were co-transfected with HPV11 E2 or HPV16 E2 plasmids and plasmids expressing STAT1, STAT2, or IRF9. Twenty hours post-transfection, cells were processed for immunofluorescence. Nuclei were stained with DAPI (blue). Scale bar, 10 μm. **(F, G)** HEK293T cells were co-transfected with plasmids expressing HPV11 E2 or HPV16 E2, along with plasmids for STAT1, STAT2, or IRF9. Co-immunoprecipitation assays were conducted 36 hours post-transfection to examine interactions between E2 and STAT1, STAT2, or IRF9. **(H)** HEK293T cells were co-transfected with HPV16 E2 and STAT1, STAT2, or IRF9 plasmids. Co-immunoprecipitation assays were conducted 36 hours post-transfection to analyze interactions among STAT1, STAT2, and IRF9.

As HPV E2 impairs IFN responses, we next investigated its impact on downstream JAK-STAT signaling. The JAK-STAT pathway is a critical downstream signaling cascade triggered by IFNs, resulting in the assembly of the ISGF3 complex (comprising STAT1, STAT2, and IRF9), which translocates to the nucleus to activate ISG transcription. RT-qPCR analysis revealed that IFN-stimulated E2-expressing cells exhibited significantly reduced mRNA levels of ISG15, ISG54, and ISG56 (**Figures 9A, B**). Activation of this pathway by IRF9-S2C (an ISGF3 activator) plasmid transfection, showed that HPV11 and HPV16 E2 further suppressed ISG expression (**Figure 9C**). Confocal microscopy confirmed that HPV11 and HPV16 E2 colocalize with STAT1, STAT2, and IRF9 (**Figures 9D, E**). Co-immunoprecipitation assays demonstrated that HPV11 and HPV16 E2 interact with STAT1, STAT2, and IRF9, thereby disrupting their interaction and inhibiting ISGF3 complex formation (**Figures 9F–H**). These findings indicate that HPV11 and HPV16 E2 suppress the JAK-STAT pathway by interfering with ISGF3 complex formation and subsequent signaling events.

### 3.7 HPV E2 prevents nuclear translocation of STAT1, STAT2, and IRF9

**Figure 10:**
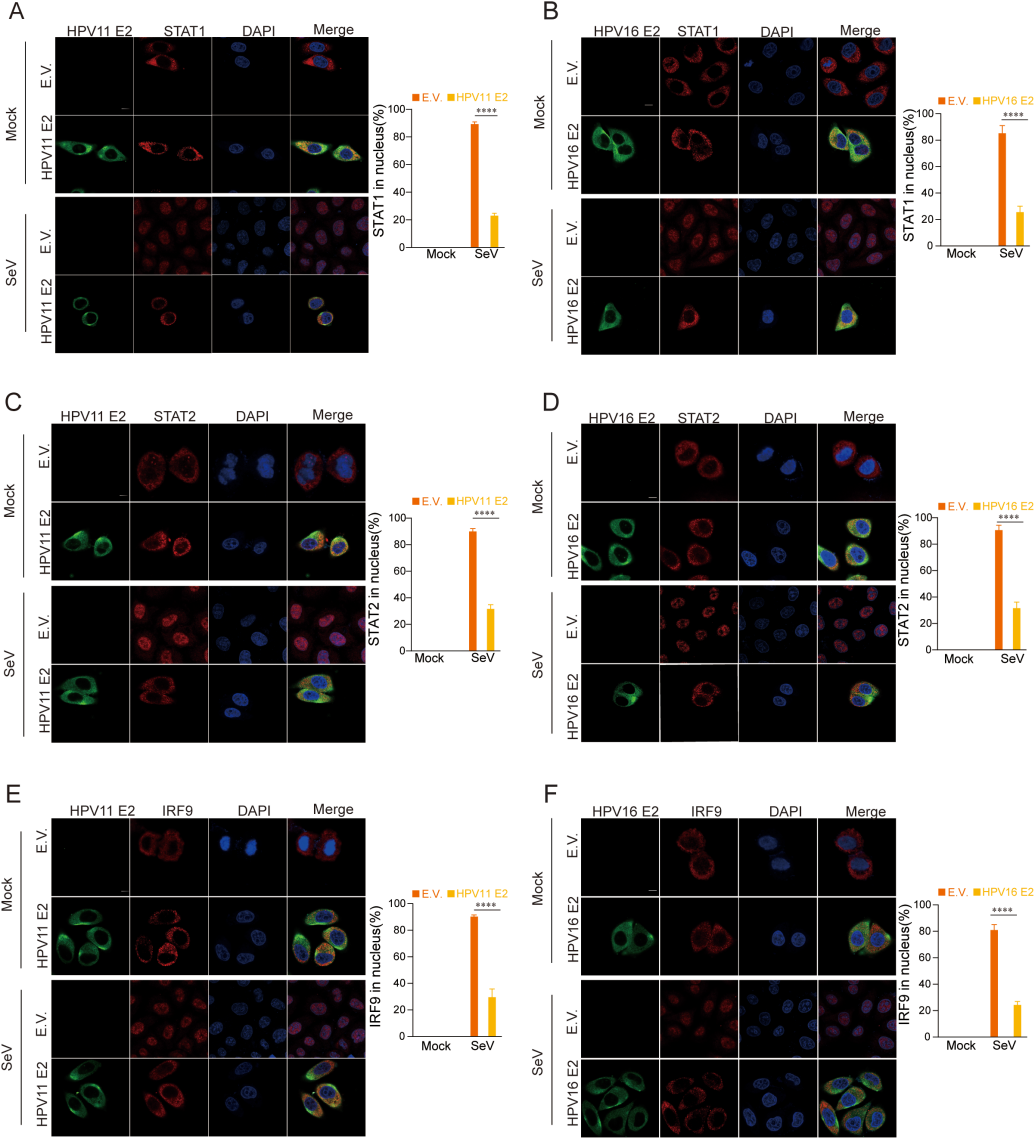
HPV11 and 16 E2 inhibit the nuclear translocation of STAT1, STAT2, and IRF9. **(A-F)** Subcellular localization of STAT1, STAT2, and IRF9. HeLa cells were co-transfected with plasmids expressing HPV11 E2 or HPV16 E2 plasmids, along with plasmids for STAT1, STAT2, or IRF9, with an empty vector serving as a control. Cells were infected with SeV (MOI=1) for 6 hours, fixed, blocked, and stained with primary antibodies followed by fluorescence-conjugated secondary antibodies. Nuclei were stained with DAPI (blue). Scale bar, 10 μm. Nuclear localization of STAT1, STAT2, and IRF9 was quantified by analyzing 40-50 cells per group, with three replicates. Statistical significance between experimental and control groups is indicated in the figure (****p < 0.0001).

Our findings indicate that HPV11 and HPV16 E2 suppress the JAK-STAT pathway by interacting the interactions between STAT1, STAT2, and IRF9, components of the ISGF3 complex that translocate to the nucleus to drive ISG expression. To evaluate the impact of HPV11 and HPV16 E2 on ISGF3 nuclear translocation, HeLa cells co-transfected with HPV E2 and STAT1, STAT2, or IRF9 were infected with SeV. Confocal microscopy revealed that in control cells, SeV infection induced nuclear translocation of STAT1, STAT2, and IRF9, while this translocation was inhibited in E2-expressing cells (**Figures 10A–F**). These results highlight the ability of HPV11 and HPV16 E2 to inhibit JAK-STAT signaling and downstream ISG transcription by preventing ISGF3 nuclear translocation.

## 4 Discussion

The HPV E2 protein has traditionally been characterized for its role in viral genome maintenance, replication, and transcription regulation. In this study, we identify HPV E2 protein as a critical modulator of host innate immune pathways, demonstrating its role in immune evasion that extends beyond its established functions in viral replication and genome maintenance. We show that HPV11 and HPV16 E2 proteins effectively suppress critical antiviral signaling pathways, including TLR3-TRIF, RIG-I/MDA5-MAVS, cGAS-STING, and JAK-STAT. This suppression results in reduced type I IFN and ISG expression, thereby fostering a microenvironment conducive to viral persistence and pathogenesis.

Transient expression of HPV16 E2 significantly inhibits IFN and ISG responses triggered by diverse viral infections and stimuli, including VSV, HSV1, MHV, and HPV16 (**Figures 1A–E**). In addition to its inhibitory effects on the IFN pathway, our study reveals that HPV E2 also suppresses NF-κB signaling, as evidenced by the downregulation of IL-6 and TNF-α. This finding suggests that E2 exerts a broader immunosuppressive role, potentially contributing to HPV persistence by dampening pro-inflammatory cytokine responses. The elevated viral titers observed in E2-expressing cells corroborate the role of E2 in immune evasion, enhancing conditions for viral persistence (**Figures 2A, B**). Luciferase reporter assays and RT-qPCR analyses further confirm that HPV E2 suppresses IFN-β and ISG expression by modulating multiple innate immune pathways, such as RIG-I/MDA5, TRIF, and STING (**Figures 3A, B**, **Figures 4A–C**). These findings are consistent with previous studies showing that HPV E2 downregulates MDA5, IFN-κ and STING, highlighting its role in attenuating innate immune signaling (42, 43).

Previous studies have demonstrated that HPV16 E6 binds IRF3, inhibiting its transcriptional activity (51), whereas HPV16 E7 promotes STING degradation via NLRX1, thereby disrupting the cGAS-STING pathway (52). HPV18 E7 similarly antagonizes DNA sensing by inhibiting the cGAS-STING pathway, and both HPV16 and HPV18 E7 activate SUV39H1-mediated epigenetic silencing of cGAS, STING, and RIG-I (34). Co-immunoprecipitation assays revealed that HPV E2 proteins target key components of innate immune signaling pathways to suppress host antiviral defenses (**Figures 5A, B**). Specifically, HPV11 and HPV16 E2 proteins bind to RIG-I, MDA5, MAVS, TRIF, and STING, disrupting the complex formation necessary for IFN induction. These interactions prevent MAVS, TRIF, and STING from associating with TBK1, thereby inhibiting TBK1 and IRF3 phosphorylation and blocking IRF3 nuclear translocation—crucial steps required for IFN production (**Figures 8A–E**). Our findings reveal that E2-mediated inhibition of IRF3 extends beyond the suppression of its phosphorylation. Although E2 reduces IRF3 phosphorylation (**Figure 8A**), IRF3-5D, a phospho-mimetic mutant, remains repressed by E2 (**Figures 3**, **4**). This strongly suggests that E2 inhibits IRF3 activation through an additional mechanism downstream of phosphorylation. Given that IRF3 activation requires its phosphorylation-dependent dissociation from adaptor complexes and subsequent nuclear translocation, our data support a model in which E2 primarily impairs IRF3 nuclear transport rather than merely blocking phosphorylation. This hypothesis is directly supported by our nuclear-cytoplasmic fractionation and immunofluorescence analyses (**Figures 8B–E**), which show that E2 prevents IRF3 from translocating into the nucleus, thereby impairing its ability to initiate IFN transcription. Mechanistically, this may involve E2 stabilizing IRF3’s interaction with upstream adaptor proteins, disrupting nuclear import machinery, or actively promoting cytoplasmic sequestration. These findings uncover a previously unrecognized mode of immune suppression by E2, highlighting its ability to target multiple stages of the IRF3 activation cascade and reinforcing its role as a key modulator of HPV-mediated immune evasion. Our findings demonstrate that HPV E2 exerts a comparable but broader role in immune suppression by targeting multiple signaling pathways. This newly identified function of HPV E2 underscores the diverse strategies HPV employs to establish persistence in host cells and evade immune detection, offering novel insights into its pathogenic mechanisms.

HPV16 E6 and E7 suppress the JAK-STAT pathway by reducing STAT1 expression and impairing its phosphorylation and nuclear translocation, thereby disrupting ISGF3 complex formation and ISG transcription (35). E6 further interferes with STAT2 activation through interactions with Tyk2, while E7 disrupts the interaction between IRF9 with STAT1/STAT2, exacerbating ISGF3 complex inhibition (37–39). In this study, we demonstrated that HPV E2 proteins disrupt the JAK-STAT pathway by directly interacting with STAT1, STAT2, and IRF9, thereby inhibiting ISGF3 complex formation and suppressing ISG expression. Using IFN-α and IFN-β to activate the JAK-STAT pathway in HEK293T cells, we observed that HPV16 E2 significantly inhibited downstream ISG expression (**Figures 9A, B**). Furthermore, employing a self-constructed IRF9-S2C plasmid to mimic ISGF3 transcriptional activation independently of IFN stimulation, we showed that E2 suppresses ISG induction by ISGF3 (**Figure 9C**). Co-immunoprecipitation and confocal microscopy assays confirmed that E2 interacts with STAT1, STAT2, and IRF9, blocking ISGF3 complex formation and nuclear translocation, further suppressing ISG transcription (**Figures 9D–H**). This inhibition curtails ISG-mediated antiviral responses, establishing E2 as a potent contributor to HPV’s immune evasion strategy, akin to the established roles of E6 and E7 in targeting IRF3 and STING. Collectively, these findings suggest that HPV employs a coordinated strategy involving multiple viral proteins, including E2, E6, and E7, to achieve robust immune suppression and sustain viral persistence. These findings highlight E2’s dual ability to suppress early PRR signaling and downstream ISG expression, adding a novel dimension to HPV’s immune evasion mechanisms. In contrast to E6 and E7, which primarily target specific immune checkpoints, E2 functions as a broad-spectrum inhibitor, suppressing multiple arms of the host’s antiviral response, including the RIG-I/MDA5-MAVS, TLR3-TRIF, cGAS-STING, and JAK-STAT pathways. This broader suppression underscores the diverse strategies employed by HPV to evade immune detection and sustain persistence. While E6 and E7 are well-established immune suppressors, our findings position E2 as an additional and critical player in HPV’s immune evasion repertoire.

Although these findings underscore E2’s role in immune evasion, further *in vivo* studies are required to validate these mechanisms in a physiological context, particularly during natural HPV infections. Moreover, the reliance on overexpression models may not fully reflect endogenous E2 levels and functions in naturally infected cells, potentially influencing the degree of immune suppression observed. However, studying HPV E2 expression and function under natural infection conditions presents significant challenges. Unlike many other viruses, HPV requires epithelial cell differentiation to complete its life cycle, making *in vitro* propagation difficult and limiting the feasibility of high-titer infection models. While organotypic raft cultures allow some level of viral replication, they yield low viral titers and are not conducive to large-scale functional studies. Additionally, patient-derived HPV-infected samples, while potentially informative, are difficult to obtain due to the virus’s restricted tropism for stratified squamous epithelium and its typically low and heterogeneous gene expression *in vivo*. Ethical and regulatory constraints further limit access to clinical specimens, complicating efforts to analyze endogenous E2 expression and colocalization with immune signaling proteins. Future research should employ *in vivo* models, such as animal systems or organotypic cultures, to more accurately replicate the HPV infection microenvironment. The use of patient-derived samples could further elucidate the role of E2 under natural infection conditions, providing clinically relevant insights into its function.

**Figure 11:**
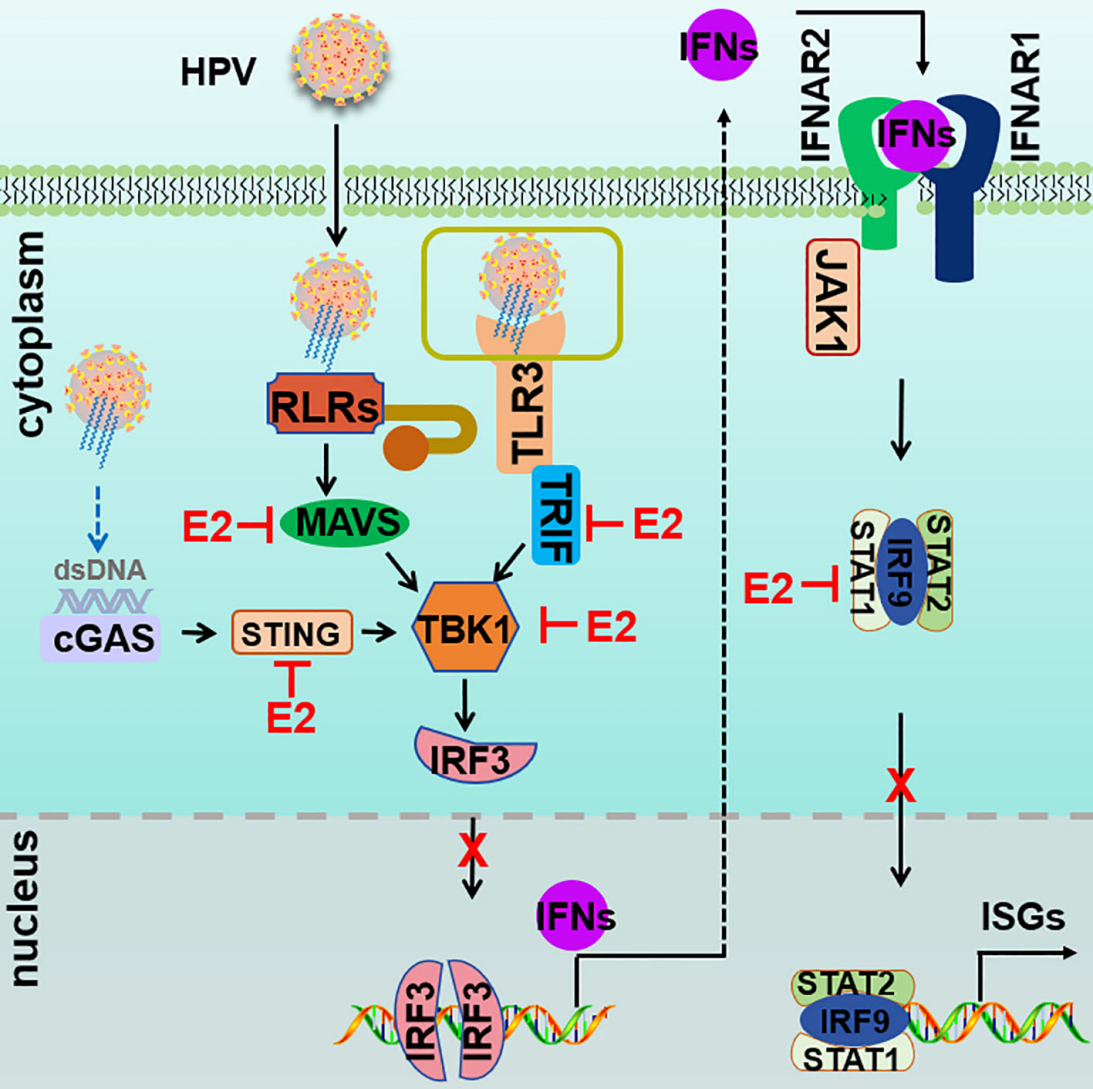
Proposed model of HPV E2-mediated suppression of innate immune signaling. Upon infection, RIG-I/MDA5-MAVS, TLR3-TRIF, cGAS-STING, and JAK-STAT pathways are activated to induce the expression of IFNs and ISGs. HPV E2 disrupts these pathways by targeting core signaling molecules, thereby inhibiting signalosome formation and suppressing of IRF3 phosphorylation and nuclear translocation. In addition, HPV E2 inhibits the JAK-STAT pathway by interacting with STAT1, STAT2, and IRF9, preventing ISGF3 complex assembly and nuclear translocation. Together, these mechanisms suppress IFN production and ISG induction, thereby dampening antiviral immunity and promoting viral replication.

This study establishes HPV16 E2 as a critical immune modulator, expanding its recognized roles in viral replication and genome maintenance to include the suppression of key antiviral pathways, including RIG-I/MDA5-MAVS, TLR3-TRIF, cGAS-STING, and JAK-STAT. Importantly, HPV16 E2 exhibits a dual role by inhibiting both upstream PRR pathways and downstream JAK-STAT signaling, establishing it as a critical factor in immune evasion (**Figure 11**). Through its modulation of these pathways, HPV16 E2 effectively suppresses IFN responses, thereby facilitating viral persistence and immune evasion. These findings provide novel insights into HPV’s immune evasion strategies, positioning E2 as a broad-spectrum inhibitor with distinct functions that complement, yet differ from, those of E6 and E7. This study not only advances our understanding of HPV pathogenesis but also identifies E2 as a promising therapeutic target for augmenting antiviral immunity in HPV-associated diseases.

## Data Availability

The original contributions presented in the study are included in the article/**Supplementary Material**. Further inquiries can be directed to the corresponding author.
