# Supplementary material for: Human papillomavirus E2 proteins suppress innate antiviral signaling pathways
Source: Front Immunol. 2025 Apr 8;16:1555629. doi: 10.3389/fimmu.2025.1555629 (PMC12011818; doi:10.3389/fimmu.2025.1555629)
Supplement: Supplementary file 1 [file Table1.docx]

**Supplementary materials**

**Human papillomavirus E2 Proteins Suppress Innate Antiviral Signaling Pathways to Facilitate Persistence**

Jin-Xin Li ^1^, Jing Zhang ^1^, Cheng-Hao Li ^2^, Qing Zhang ^3, 4^, Beihua Kong ^3, 4^, Pei-Hui Wang ^1, 2, *^

^1^ Department of Infectious Disease and Hepatology, The Second Hospital of Shandong University, Cheeloo College of Medicine, Shandong University, Jinan, 250033, Shandong, China;

^2^ Key Laboratory for Experimental Teratology of Ministry of Education and Advanced Medical Research Institute, Cheeloo College of Medicine, Shandong University, Jinan, Shandong 250012, China.

^3^ Department of Obstetrics and Gynecology, Qilu Hospital, Shandong University, 107 Wenhua Xi Road, Jinan, 250012, China.

^4^ Gynecologic Oncology Key Laboratory of Shandong Province, Qilu Hospital, Shandong University, Jinan, 250012, China.

*Correspondence: [pei-hui.wang@connect.hku.hk](mailto:pei-hui.wang@connect.hku.hk)

**Supplementary Table S1. Primers used in this study**

| Primer name | Sequence (5′-3′) | Usage |
| --- | --- | --- |
| GAPDH-F  GAPDH-R | GGAGCGAGATCCCTCCAAAAT  GGCTGTTGTCATACTTCTCATGG | RT-qPCR |
| IFN-β-F  IFN-β-R | TTGCTCTCCTGTTGTGCTTC  AAGCCTCCCATTCAATTGCC | RT-qPCR |
| ISG56-F  ISG56-R | CTAAGCAAAACCCTGCAGAAC  TCAGGCATTTCATCGTCATC | RT-qPCR |
| ISG54-F  ISG54-R | CGAACAGCTGAGAATTGCAC  TTCTCCCTCCATCAAGTTCC | RT-qPCR |
| CXCL10-F  CXCL10-R | GTGGCATTCAAGGAGTACCTC  GACCTTTCCTTGCTAACTGCT | RT-qPCR |
| ISG15-F | AGGCAGCGAACTCATCTTTG | RT-qPCR |
| ISG15-R | GGACACCTGGAATTCGTTG |  |
| IL6-F | TTCGGTCCAGTTGCCTTCT | RT-qPCR |
| IL6-R | TGCCTCTTTGCTGCTTTCA |  |
| TNFα-F | AGCCCATGTTGTAGCAAACC | RT-qPCR |
| TNFα-F | AGGACCTGGGAGTAGATGAGG |  |
| HPV16 E2-F | CTTGGTACCGAGCTCGCCACCATGGAGACCCTGTGCCAGAGG | ORF cloning |
| HPV16 E2-R | CACGGTGTTGTCCTTTCTAGAGATGCTCATGAAGCCGGTGCT |  |
| HPV11 E2-F | CTTGGTACCGAGCTCGCCACCATGGAGGCCATCGCCAAGAG | ORF cloning |
| HPV11 E2-R | CACGGTGTTGTCCTTTCTAGACAGCAGGTGCAGGCTCATGA |  |

F: forward primer. R: reverse primer.
